# ﻿Two new species of the genus *Symphylella* (Symphyla, Scolopendrellidae) from China and the significance of the frons chaetotaxy

**DOI:** 10.3897/zookeys.1138.96424

**Published:** 2023-01-05

**Authors:** Ya-Li Jin, Yun Bu

**Affiliations:** 1 Natural History Research Center, Shanghai Natural History Museum, Shanghai Science & Technology Museum, Shanghai, 200041, China Shanghai Natural History Museum Shanghai China

**Keywords:** Chaetotaxy, frons, mandible, Myriapoda, taxonomy

## Abstract

*Symphylellamacrochaeta***sp. nov.** and *Symphylellalongispina***sp. nov.** from China are described and illustrated. *Symphylellamacrochaeta***sp. nov.** is characterized by 10 extremely long macrosetae arranged as 4/4/2 on the frons, tergites with broad triangular processes, and 4+4 setae on the first tergite. *Symphylellalongispina***sp. nov.** is characterized by a thick and prominent labrum, distinctly long proximal spines on the mandible, eight macrosetae arranged as 4/2/2 on frons, 3+3 setae on first tergite, and narrow triangular processes on the tergites. Detailed comparisons of the new species with similar species are presented. In addition, the frons chaetotaxy of *Symphylella* is illustrated and discussed for the first time and proposed as a significant diagnostic character for the taxonomic study of the genus.

## ﻿Introduction

Symphylans are minute soil arthropods present in various habitats, and some species are important crop pests (Chau 2015; Jin and Bu 2022). However, the diversity of symphylans in China is poorly known, with only eight species recorded until now (Bu and Jin 2018; Jin and Bu 2018, 2019, 2020; Jin et al. 2019). *Symphylella* Silvestri, 1902 is a diverse group of symphylans with 51 species described worldwide (Bu and Jin 2018; Jin et al. 2019; Jin and Bu 2020), but only four have been recorded in China: *S.macropora* Jin & Bu, 2019 and *S.zhongi* Jin & Bu, 2019 from Tibet (Jin et al. 2019), and *S.communa* Jin & Bu, 2020 and *S.minuta* Jin & Bu, 2020 from East China (Jin and Bu 2020). During the last five years, the symphylan fauna from Xinjiang, Zhejiang and Shanghai was investigated and two new species of *Symphylella* were identified. They are described in the present paper. The frons chaetotaxy of the six Chinese species of *Symphylella* is compared in detail.

## ﻿Materials and methods

Specimens were obtained by extraction of soil and litter samples from broad-leaf and bamboo forests using Berlese-Tullgren funnels. Specimens were preserved in 80% ethanol. They were mounted on slides using Hoyer’s solution and dried in an oven at 50 °C. Observations were performed under a phase contrast microscope (Leica DM 2500). Photographs were taken with a digital camera (Leica DMC 4500) mounted on the microscope. Line drawings were made using a drawing tube. All specimens are deposited in the collections of Shanghai Natural History Museum (**SNHM**), Shanghai, China.

## ﻿Results

### ﻿Taxonomy

**Family Scolopendrellidae Bagnall, 1913**

#### ﻿Genus Symphylella

Taxon classificationAnimaliaTetramerocerataScolopendrellidae

Silvestri, 1902

FAB2C005-016A-5BD5-BE1A-11C29C95DF73

##### Type species.

*Symphylellaisabellae* (Grassi, 1886), described from Italy.

#### ﻿ Symphylella macrochaeta

Taxon classificationAnimaliaTetramerocerataScolopendrellidae

Jin & Bu sp. nov.

BF3EEFD2-88CE-5613-BEDF-B69743567C73

https://zoobank.org/EDC04E98-38F3-43BD-A6F0-927F77D4A12C

**Figure 1.**
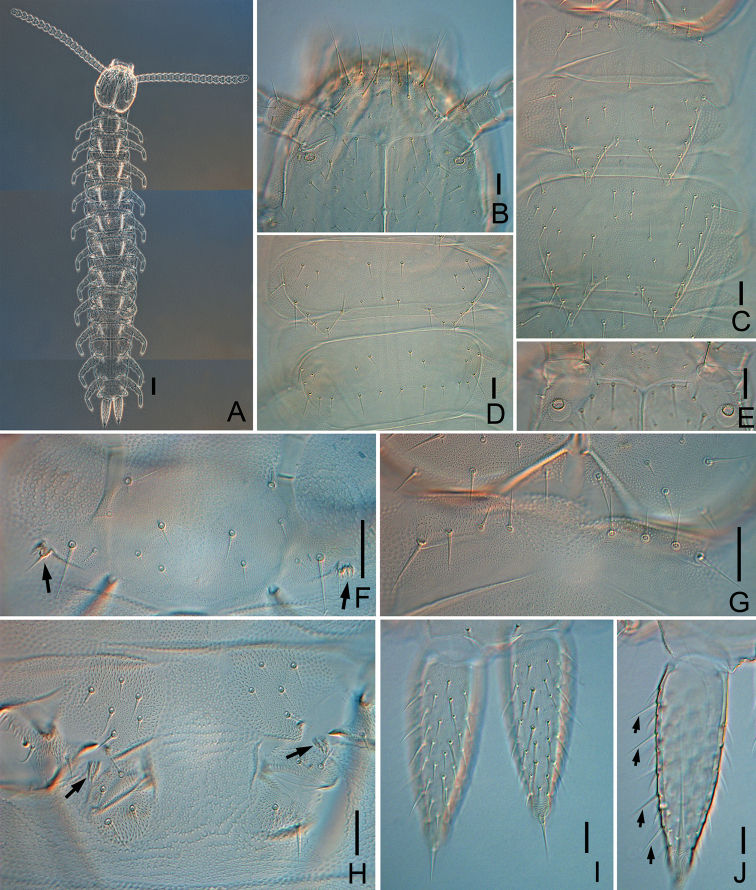
*Symphylellamacrochaeta* sp. nov. **A** habitus, dorsal view **B** head, anterior part, dorsal view **C** tergites 1–3 **D** tergites 13–14 **E** Tömösváry organ **F** first pair of legs (arrows indicate reduced legs) **G** tergite 1 **H** styli and coxal sacs on base of leg 3 (arrows indicate styli). **I** cerci, dorsal view **J** left cercus, ventral view (arrows indicate long and erect outer setae). Scale bars: 100 μm (**A**); 20 μm (**B–J**).

**Figure 2.**
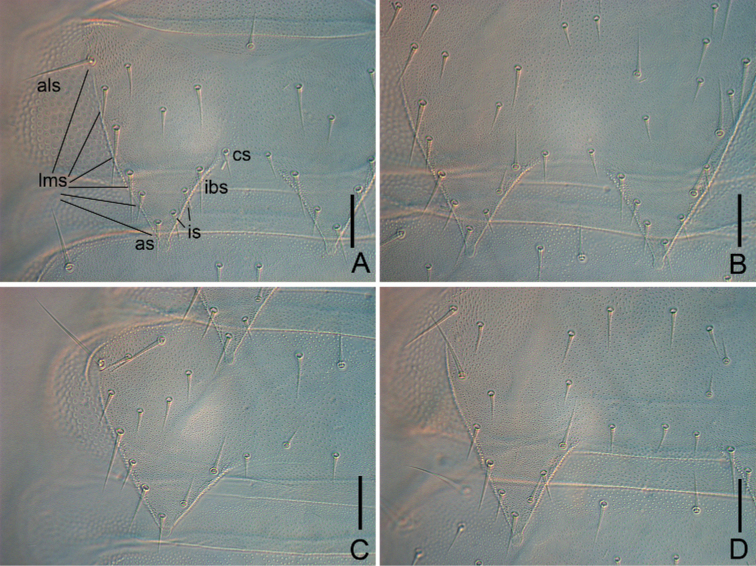
*Symphylellamacrochaeta* sp. nov. **A** tergite 2 (*als* – anterolateral seta, *lms* – lateromarginl setae, *as* – apical seta, *is* – inserted seta, *ibs* – inner basal seta, *cs* – central seta) **B** tergite 3 **C** tergite 4, left side **D** tergite 5, left side. Scale bars: 20 μm.

**Figure 3.**
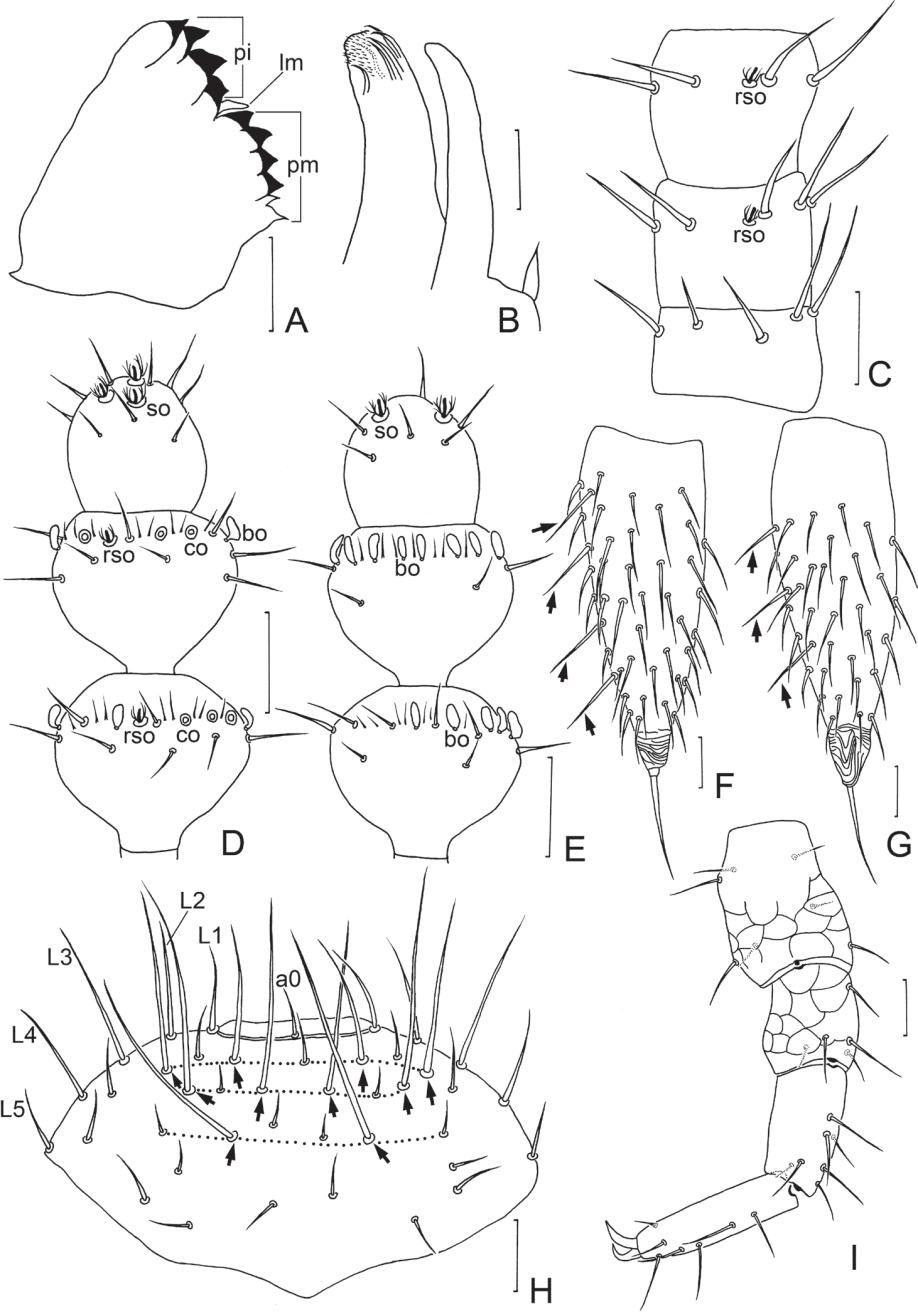
*Symphylellamacrochaeta* sp. nov. **A** mandible, lateral view (*pi* – pars incisivus, *pm* – pars molaris, *lm* – lacinia mobilis) **B** first maxilla **C** left 1–3 antennomere, dorsal view **D** terminal three antennomeres, dorsal view (*bo* – bladder-shaped organ, *co* – cavity-shaped organ, *rso* – rudimentary spined sensory organ, *so* – spined sensory organ) **E** terminal three antennomeres, ventral view **F** left cercus, dorsal view (arrows indicate long and erect outer setae) **G** left cercus, ventral view **H** frons (L1–L5 – lateral setae, a0 – antero-central seta, arrows indicate macrosetae) **I** leg12, dorso-lateral view. Scale bars: 20 μm.

Figs 1 , 2 , 3 , Tables 1 , 2 , 3

##### Diagnosis.

*Symphylellamacrochaeta* sp. nov. is characterized by 10 extremely long macrosetae arranged as 4/4/2 on the frons, 4+4 setae on the first tergite and broad triangular processes on tergites.

##### Material examined.

***Holotype***: female (slide no. ZJ-ZS-SY2020029) (SNHM), China, Zhejiang Province, Zhoushan City, Changgang Mountain Forest Park, extracted from soil samples of broad-leaf forest, alt. 250 m, 30°2'N, 121°7'E, 17-XI-2020, coll. Y. L. Jin et al.

***Paratypes***: 10 females (slides no. ZJ-ZS-SY2020006, ZJ-ZS-SY2020008, ZJ-ZS-SY2020014–ZJ-ZS-SY2020016, ZJ-ZS-SY2020024–ZJ-ZS-SY2020028) (SNHM), same data as holotype. 2 females (slides no. SH-JZGY-SY2017032, SH-JZGY-SY2017034), China, Shanghai, Jiuzi Park, extracted from soil and litter samples of bamboo forest, alt. 14 m, 31°15'N, 121°28'E, 25-V-2017, coll. Y. L. Jin.

***Non-type specimens***: 18 juveniles with 7–10 pairs of legs, same data as holotype; 5 juveniles with 9 or 10 pairs of legs, China, Shanghai, Jiuzi Park, extracted from soil and litter samples of bamboo forest, alt. 14 m, 31°15'N, 121°28'E, 25-V-2017, coll. Y. L. Jin; 1 juvenile with 10 pairs of legs, China, Shanghai, Tianma Mountain, extracted from soil samples of bamboo forest, alt. 98 m, 31°5'N, 121°9'E, 10-V-2017, coll. Y. Bu.

##### Description.

Adult body 2.1 mm long in average (1.9–2.2 mm, *n* = 11), holotype 2.1 mm (Fig. 1A).

***Head*** length 250–280 μm, width 223–265 μm, with widest part on equal level of points of articulation of mandibles. Central rod distinct in both anterior (65–70 μm) and posterior (75–85 μm) parts, with an obvious middle node-like interruption. Head dorsally covered with setae of different lengths (Fig. 1B). Frons with 5+5 lateral setae, 10 extremely long macrosetae (58–73 μm) arranged as 4/4/2 (counted from anterior row to posterior row) and 4–5.6 times as long as antero-central seta (a0) (Fig. 3H), and 20–21 short to medium-length setae (8–16 μm) (Figs 1B, 3H). Cuticle on anterolateral part of head with coarse granules (Fig. 1B).

***Tömösváry organ*** globular, diameter 15–20 μm, about half of greatest diameter of third antennomere (35–40 μm), opening round (9–12 μm), inner margins of opening covered with regular vertical striae (Fig. 1E).

***Mouthparts*.** Mandible composed by pars incisivus (*pi*) and pars molaris (*pm*), with movable appendage lacinia mobilis (*lm*) inserted between them. Pars incisivus with 4 distinct thick teeth, pars molaris with 4 smaller teeth and 2 proximal spines, and lacinia mobilis with only 1 blunt process observed from lateral view (Fig. 3A). First maxilla has 2 lobes, inner lobe with 6 hook-shaped teeth and pubescent apically, palp pointed (Fig. 3B). Anterior part of second maxilla with many small protuberances, each carrying 1 seta, distal setae thicker and spiniform; posterior part with sparse setae. Cuticle of second maxilla covered with dense pubescence.

***Antennae*** with 16–20 antennomeres (18 in holotype), about 0.2 of body length. First antennomere cylindrical, length about 0.5–0.8 of greatest diameter (width 33–40 μm, length 18–25 μm), with 6 or 7 setae in 1 whorl, longest inner seta 16–18 μm (Fig. 3C). Second antennomere wider (35–38 μm) than long (27–33 μm), with 8 setae evenly inserted around antennal wall with interior setae (15 μm) slightly longer than exterior ones (11 μm) (Fig. 3C). Chaetotaxy of third antennomere similar to preceding ones. Setae on proximal antennomeres longer and on distal antennomeres shorter. Proximal antennomeres with only primary whorl of setae (Fig. 3C). Secondary whorl appearing ventrally on antennomeres 6–8. Four kinds of sensory organs observed on antenna: rudimentary spined sensory organs (*rso*) on dorsal side of most antennomeres (Fig. 3C, D); spined sensory organs (*so*) with more surrounding spines and larger than *rso*, only present on apical antennomere (Fig. 3D, E); cavity-shaped organs (*co*) on antennomeres 6 and 7 next to subapical one (Fig. 3D); bladder-shaped organs (*bo*) on antennomeres 9–11 next to subapical one increasing in number on subdistal antennomeres to 15 in maximum (Fig. 3D, E). Apical antennomere subspherical, with its length as long as width (28–30 μm), with 5 spined sensory organs consisting of 3 or 4 curved spines around a central pillar and 12–16 setae located distally (Fig. 3D, E). All antennomeres covered with short pubescence. Chaetotaxy and sensory organs on antennae of holotype are given in Table 1.

**Table 1.** Numbers of setae and sensory organs on antennae of *Symphylellamacrochaeta* sp. nov. (holotype).

Antennomere	Primary whorl setae	Secondary whorl setae	Rudimentary spined sensory organs	Cavity-shaped organs on dorsal side	Bladder-shaped organs
1	6		1		
2	8		1		
3	8		1		
4	9		1		
5	9		1		
6	11		1	1	
7	11	2	1	1	
8	11	2	0	1	
9	10	3	0	1	
10	11	3	1	1	1
11	11	4	1	1	1
12	11	5	1	1	1
13	11	6	1	1	2
14	11	6	0	1	3
15	10	7	0	1	3
16	10	6		3	5
17	10	6		3	8

***Trunk*** with 17 tergites. Tergites 2–13, and 15 each with 1 pair of triangular processes. Length from base to tip of processes somewhat shorter than or same as its basal width; basal distance between processes longer than their length from base to tip except on tergites 2 and 3 (Table 3). All processes with roundish swollen ends (Figs 1C, 2A–D). Definition of chaetotaxy on tergite as follow: anterolateral setae (*als*) located on anterolateral angle of each tergite; apical seta (*as*) most close to process apex; lateromarginl setae (*lms*) located on lateral margin of process and including *als* and *as*; inner basal setae (*ibs*) located on inner base of process; inserted setae (*is*) present between *ibs* and *as*; central setae (*cs*) present between base of processes; other setae including all setae except above nominated ones (Fig. 2A). Anterolateral setae of tergites 2, 3, 5, 6, 8, 9 11 and 12 slightly shorter than length of process of same tergite, that of tergites 4, 7, 10, 13 and 15 subequal or slightly longer than length of process of same tergite. Processes with 1 or 2 inserted setae. All tergites pubescent (Fig. 2A–D).

***Tergites*.** Tergite 1 reduced, with 4+4 setae of different length (Fig. 1G). Tergite 2 complete, with 2 broad triangular posterior processes, 6 or 7 lateromarginal setae, 1 or 2 inserted setae, 1 or 2 central setae (Table 2), with anterolateral setae 0.8–0.9 time as long as length of process, length of processes 0.8–1.0 time as long as broad, basal distance between processes 0.6–0.9 time as long as their length (Figs 1C, 2A). Tergite 3 complete, broader and longer than preceding one, with ratios of 0.7–0.9, 0.8–1.0, and 0.6–0.9 respectively, 8–10 lateromarginal setae, 1 or 2 inserted setae, 1–3 central setae (Figs 1C, 2B). Tergite 4 broader than tergite 3, with ratios 1–1.3, 0.6–0.7, and 1.3–2.5 respectively, 5–7 lateromarginal setae (Fig. 2C). Chaetotaxy of tergites 5–7, 8–10, and 11–13 similar as tergites 2–4 (Fig. 2D). Pattern of alternating tergite lengths of 2 short-tergites followed by long-tergite only disrupted at caudal end (Table 3). Tergites 14 and 16 without processes and with 17–26 and 12–17 setae respectively (Fig. 1D). Tergite 17 with 27–38 setae. Chaetotaxy and measurements of tergites are given in Tables 2 and 3.

**Table 2.** Chaetotaxy of tergites of *Symphylellamacrochaeta* sp. nov. (holotype in brackets).

Tergite	Lateromarginal setae	Inserted setae	Central setae	Other setae
1	4+4			
2	6–7 (6)	1–2 (2)	1–2 (2)	6–10 (6)
3	8–10 (8)	1–2 (2)	1–3 (2)	14–25 (14)
4	5–7 (6)	1–2 (1)	3–5 (3)	10–15 (11)
5	5–7 (6)	1–2 (2)	2–4 (3)	7–13 (12)
6	8–10 (9)	1–2 (2)	2–4 (4)	17–28 (20)
7	5–7 (6)	1–2 (2)	4–6 (4)	10–14 (11)
8	5–7 (6)	1–3 (2)	3–5 (4)	10–14 (10)
9	8–10 (9)	1–3 (1)	3–5 (3)	20–27 (22)
10	5–6 (6)	1–2 (1)	4–6 (4)	9–14 (11)
11	5–8 (6)	1–2 (1)	3–5 (4)	7–14 (9)
12	7–10 (8)	1–2 (2)	3–5 (3)	15–24 (15)
13	4–7 (5)	0–2 (1)	2–5 (3)	8–14 (8)
14				17–26 (21)
15	6–9 (7)	0/2 (0)	2–4 (3)	14–19 (14)
16				12–17 (14)
17				27–38 (29)

**Table 3.** Measurements of tergites and processes of *Symphylellamacrochaeta* sp. nov. (mean ± se, *n* = 11, in μm) (holotype in brackets).

Tergite	Length	Width	Length of processes	Basal width of processes	Basal distance between processes
1	24 ± 7 (23)	141 ± 13 (138)			
2	48 ± 5 (45)	144 ± 7 (150)	37 ± 3 (33)	41 ± 3 (43)	28 ± 4 (32)
3	102 ± 14 (100)	186 ± 25 (180)	41 ± 4 (42)	45 ± 3 (47)	33 ± 3 (37)
4	57 ±7 (55)	194 ± 17 (205)	33 ± 3 (35)	49 ± 5 (52)	60 ± 8 (65)
5	71 ± 12 (65)	183 ± 10 (190)	41± 5 (45)	43 ± 4 (45)	62 ± 7 (67)
6	121 ± 9 (125)	223 ± 38 (235)	47± 5 (47)	48 ± 4 (47)	62 ± 6 (67)
7	70 ± 9 (65)	229 ± 18 (242)	36 ± 5 (40)	48 ± 6 (50)	85 ± 10 (95)
8	74 ± 5 (82)	204 ± 13 (205)	45 ± 4 (50)	45 ± 3 (50)	77 ± 10 (85)
9	114 ± 26 (120)	253 ± 21 (250)	46 ± 4 (50)	46 ± 4 (50)	72 ± 6 (75)
10	76 ± 14 (82)	235 ± 23 (250)	34 ± 3 (37)	48 ± 4 (50)	92 ± 16 (100)
11	73 ± 7 (70)	207 ± 13 (210)	41 ± 4 (45)	43 ± 3 (42)	78 ± 9 (85)
12	115 ± 7 (115)	255 ± 12 (260)	41 ± 4 (45)	47 ± 7 (50)	74 ± 9 (77)
13	68 ± 10 (60)	233 ± 24 (245)	28 ± 5 (32)	48 ± 6 (55)	89 ± 12 (90)
14	68 ± 10 (60)	205 ± 20 (210)			
15	93 ± 9 (90)	226 ± 21 (247)	28 ± 3 (32)	45 ± 5 (52)	67 ±8 (75)
16	72 ± 6 (80)	185 ± 25 (200)			
17	110 ± 8 (125)	172 ± 21 (175)			

***Legs*.** First pair of legs reduced to 2 small hairy cupules, each with 1 long seta (9–10 μm) (Fig. 1F). Basal areas of legs 2–12 each with 3–8 setae (Fig. 1H). Leg 12 0.8–0.9 time as long as length of head (Fig. 3I), trochanter 1.1–1.2 times as long as wide (50–75 μm, 41–67 μm), with 7 setae; femur almost as long as wide (35–40 μm, 30–40 μm), with 5 setae and dorsal protruding longest setae (18–25 μm) about 0.6 time of greatest diameter of podomere; tibia nearly1.6–1.9 times longer than wide (45–55 μm, 25–30 μm), with 5 dorsal setae: 3 straight and protruding, 2 slightly curved and depressed, longest setae 0.7–1.0 of greatest diameter of tibia, 2 ventral setae distinctly shorter than dorsal ones; tarsus cylindrical, about 3–4.3 times as long as wide (58–75 μm, 16–19 μm) with 6 dorsal setae: 3 or 4 straight and protruding, others curved and depressed, longest setae (15–22 μm) same with greatest width of podomere, 1 ventral seta close to claw distinctly shorter than dorsal ones. Claws curved, anterior one somewhat broader than posterior one, posterior one more curved than former. Trochanter and femur with cuticular thickenings in pattern of large scales laterally (Fig. 3I). All legs covered with dense pubescence except areas with cuticular thickenings.

***Coxal sacs*** present at bases of legs 3–9, fully developed, each with 4 or 5 setae on surface (Fig. 1H). Corresponding area of leg 2, 10, 11, and 12 replaced by 2–4 setae respectively.

***Styli*** present at base of legs 3–12, slender (length 6–9 μm, width 4–6 μm), basal part with dense straight hairs; distal quarter hairless and with blunt apex (3–5 μm) (Fig. 1H).

***Sense calicles*** located on 2 ventral protuberances of last tergite, posterior to base of leg 12, with smooth margin around pit. Sensory seta inserted in cup center, extremely long (110–140 μm).

***Cerci*** about 0.5–0.6 of head length, 2.5–3 times as long as its greatest width (125–170 μm, 50–63 μm), densely covered with 75–90 subequal setae (Figs 1I–J, 3F–G). Two types of setae inserted on cercus: 7 and 8 long and erect setae located in outer side, and others slightly curved and depressed. Longest outer seta (25–30 μm) 0.4–0.6 of greatest width of cerci (Figs 1J, 3F–G), terminal area short (25–30 μm), circled by 9 layers of curved ridges. Terminal setae (25–32 μm) almost as long as terminal area (Figs 1I, 3F–G).

##### Etymology.

From the Greek words “macro” meaning “large” and “chaeta” meaning “seta”. The species name “macrochaeta” is feminine and refers to extremely long setae on the frons.

##### Distribution.

China (Shanghai, Zhejiang).

##### Remarks.

*Symphylellamacrochaeta* sp. nov. has 10 extremely long macrosetae on the frons, which can distinguish it from all other congeners. It is similar to *S.communa* from East China and *S.asiatica* Scheller, 1971 from India and Sri Lanka in the shapes of the central rod, tergites, and leg 12, but the new species differs in the chaetotaxy of the first tergite (4+4 setae in *S.macrochaeta* sp. nov. and *S.communa* vs 3+3 setae in *S.asiatica*) and in the shape of stylus (slender in *S.macrochaeta* sp. nov. vs subconical in *S.communa* and conical in *S.asiatica*). The new species can also be compared to *S.macropora* from Tibet in the shape of tergites and processes, but it can be easily separated by the shape and the size of the opening of the Tömösváry organ (moderate and round in *S.macrochaeta* sp. nov. vs large and elongate in *S.macropora*).

#### ﻿ Symphylella longispina

Taxon classificationAnimaliaTetramerocerataScolopendrellidae

Jin & Bu sp. nov.

D3603436-6646-537D-9651-5C0634E8BEDA

https://zoobank.org/C577A20B-B66D-43DE-8D99-339661E7374E

**Figure 4.**
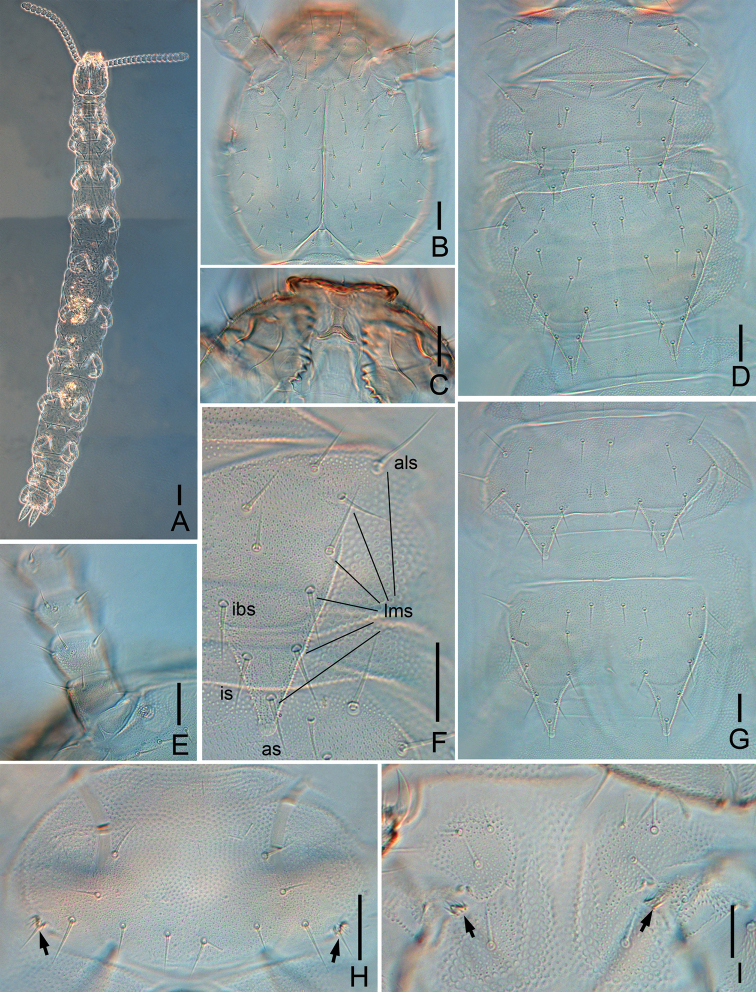
*Symphylellalongispina* sp. nov. **A** habitus, dorsal view **B** head, dorsal view **C** labrum and mandible **D** tergites 1–3 **E** Tömösváry organ and antennomeres 1–4 **F** tergite 2, right side (*als* – anterolateral seta, *lms* – lateromarginl setae, *as* – apical seta, *is* – inserted seta, *ibs* – inner basal seta) **G** tergites 4–5 **H** first pair of legs (arrows indicate the reduced legs) **I** styli and coxal sacs on base of leg 12 (arrows indicate styli). Scale bars: 100 μm (**A**); 20 μm (**B–I**).

**Figure 5.**
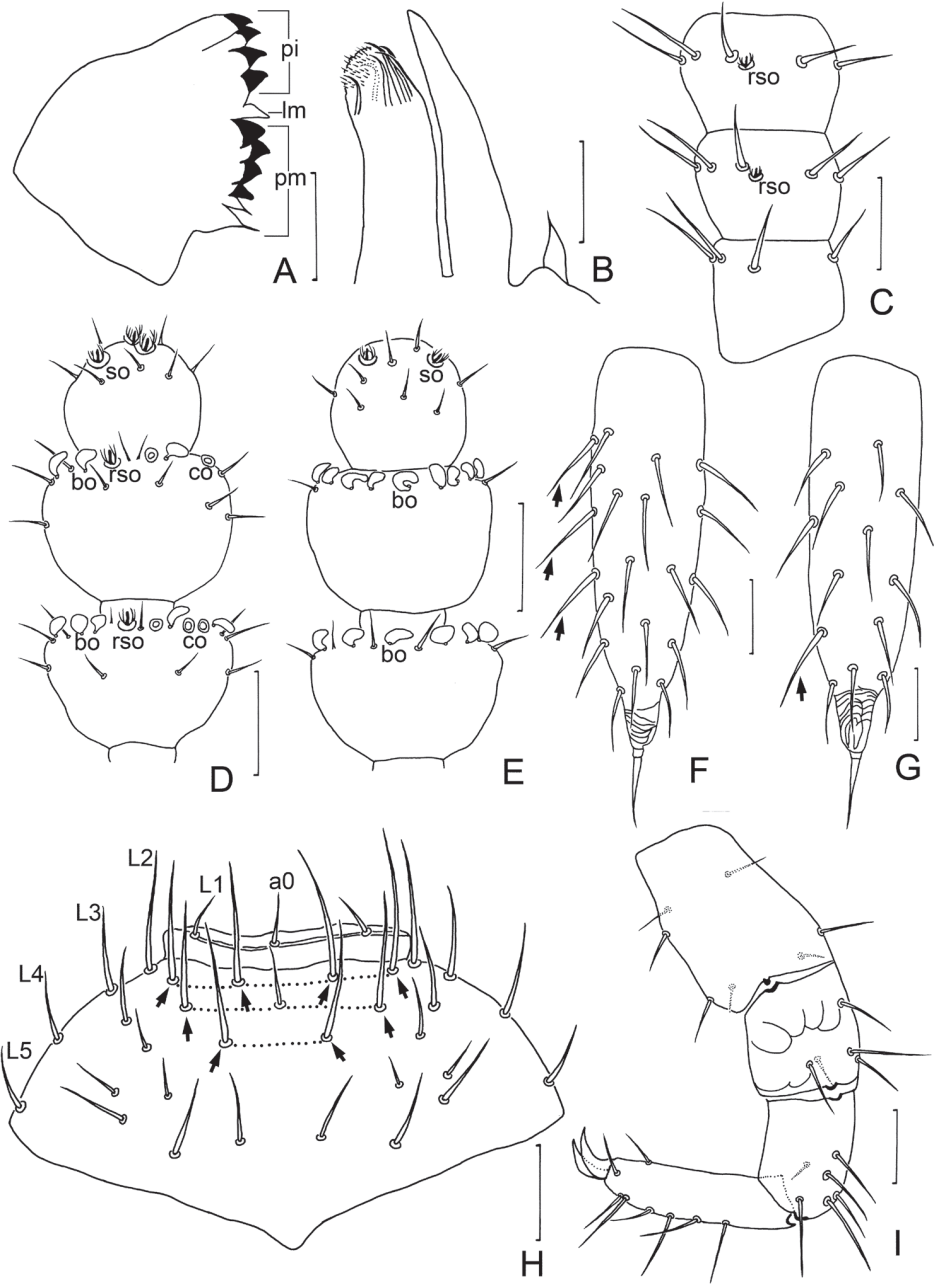
*Symphylellalongispina* sp. nov. **A** mandible, lateral view (*pi* – pars incisivus, *pm* – pars molaris, *lm* – lacinia mobilis) **B** first maxilla **C** left 1–3 antennomeres, dorsal view **D** terminal three antennomeres, dorsal view (*bo* – bladder-shaped organ, *co* – cavity-shaped organ, *rso* – rudimentary spined sensory organ, *so* – spined sensory organ) **E** terminal three antennomeres, ventral view **F** left cercus, dorsal view (arrows indicate long and erect outer setae) **G** left cercus, ventral view **H** frons (L1–L5 – lateral setae, a0 – antero-central seta, arrows indicate macrosetae) **I** leg 12, dorso-lateral view. Scale bars: 20 μm.

Figs 4 , 5 , Tables 4 , 5 , 6

##### Diagnosis.

*Symphylellalongispina* sp. nov. is characterized by apparently thickened labrum, distinctly long proximal spines on the pars molaris of the mandible, eight macrosetae arranged as 4/2/2 on the frons, 3+3 setae on the first tergite and narrow triangular processes on tergites.

##### Material examined.

***Holotype***: female (slide no. XJ-SY20160003) (SNHM), China, Xinjiang, Bole City, Hariturege National Forest Park, extracted from soil samples from the forest of *Populuseuphratica*, alt. 1125 m, 40°08'N, 81°46'E, 31-VIII-2016, coll. C. W. Huang.

***Paratypes***: 5 females (slides no. XJ-SY20160001, XJ-SY20160002, XJ-SY20160004, XJ-SY20160005, XJ-SY20160006) (SNHM), same data as holotype.

##### Description.

Adult body 2.4 mm long in average (1.8–2.6 mm, *n* = 6), holotype 2.4 mm (Fig. 4A).

***Head*** length 210–225 μm, width 190–225 μm, with widest part on equal level of points of articulation of mandibles. Central rod well developed but thin, divided into 2 portions by node-like sub-median interruption, with anterior 48–50 μm and posterior 60–70 μm. (Fig. 4B). Dorsal side of head moderately covered with setae of different lengths. Frons with 5+5 lateral setae, 8 macrosetae (23–28 μm) arranged as 4/2/2 and 2.8–3.7 times as long as antero-central seta (a0) (Fig. 5H), and 16 moderate setae (14–17 μm) (Figs 4B, 5H). Cuticle on anterolateral part of head with coarse granules (Fig. 4B).

***Tömösváry organ*** globular, diameter 12–16 μm, shorter than half of greatest diameter of third antennomere (33–35 μm), opening small and round (4–6 μm), with distinct vertical inner striae (Fig. 4B, E).

***Mouthparts***. Labrum apparently thickened and protruding (Figs 4C, 5H). Mandible similar to *S.macrochaeta* sp. nov., but pars molaris with extremely long proximal spines (Figs 4C, 5A). First maxilla has 2 lobes, inner lobe with 6 hook-shaped teeth and pubescent apically, palp pointed and slightly incurved (Fig. 5B). Anterior part of second maxilla with many small protuberances, each carrying 1 seta, distal setae thick; posterior part with sparse setae. Cuticle of second maxilla covered with dense pubescence.

***Antennae*** with 16–20 antennomeres (holotype with 18), about 0.2 of body length. First antennomere cylindrical, almost same as wide as long (width 24–28 μm, length 25–28 μm), with 5–7 setae in 1 whorl, longest inner seta 14–15 μm (Figs 4E, 5C). Second antennomere wider (29–33 μm) than long (24–25 μm), with 8 setae evenly inserted around antennal wall with interior setae (15 μm) slightly longer than exterior ones (11 μm) (Figs 4E, 5C). Chaetotaxy of third antennomere similar to preceding ones. Setae on proximal antennomeres longer and on distal antennomeres shorter. Proximal antennomeres with only primary whorl of setae, in middle and subapical antennomeres with several minute setae in secondary whorl. Four kinds of sensory organs observed on antenna: rudimentary spined sensory organs on dorsal side of most antennomeres except first antennomere (Fig. 5C, D); spined sensory organs only present on terminal antennomere (Fig. 5D, E); cavity-shaped organs on antennomeres 10 and 11 next to apical one, increasing in number to 4 in maximum (Fig. 5D); bladder-shaped organs irregular, round, oval or curved, present on antennomeres 5 and 6 next to apical one increasing in number on subdistal antennomeres to 13 in maximum (Fig. 5D, E). Apical antennomere subspherical, somewhat wider than long (width 25–28 μm, length 15–20 μm), five spined sensory organs consisting of 3 or 4 curved spines around a central pillar and 13–17 setae on distal half (Fig. 5D, E). All antennomeres covered with short pubescence. Chaetotaxy and sensory organs of antennae of holotype are given in Table 4.

**Table 4.** Numbers of setae and sensory organs on antennae of *Symphylellalongispina* sp. nov. (holotype).

Antennomere	Primary whorl setae	Secondary whorl setae	Rudimentary spined sensory organs	Cavity-shaped organs on dorsal side	Bladder-shaped organs
1	6				
2	8		1		
3	8		1		
4	9		1		
5	10		1		
6	10		1		
7	10		1	1	
8	10		0	1	
9	10		0	1	
10	10	1	0	1	
11	10	1	1	1	
12	11	3	1	1	1
13	11	4	0	1	3
14	11	4	0	1	5
15	11	5		2	9
16	11	4		3	13
17	10	5		2	13

***Trunk*** with 17 tergites. Tergites 2–13 and 15 each with 1 pair of triangular processes. Length from base to tip of processes slightly longer than its basal width except for tergites 4, 7, 10 and 13, in which processes almost as broad as long; basal distance between processes of tergites distinctly longer than their length from base to tip (Table 5). All processes with distinct rounded end-swellings (Fig. 4D, F, G). Anterolateral setae of tergites 2, 3, 4, 6, 7, 9 and 10 distinctly longer than other lateromarginal setae, that of tergites 5, 8, 11–13 and 15 subequal or slightly shorter than longest ones of other lateromarginal (Fig. 4D, F, G). Anterolateral setae of tergites shorter than or subequal to process of same tergite. Processes with 1 inserted seta (*is*) (Fig. 4F). All tergites pubescent (Fig. 4F).

**Table 5.** Chaetotaxy of tergites of *Symphylellalongispina* sp. nov. (holotype in brackets).

Tergite	Lateromarginal setae	Inserted seta	Central setae	Other setae
1	3+3			
2	5–6 (6)	1 (1)	1 (1)	5–7 (6)
3	7–8 (7)	1 (1)	1 (1)	16–19 (16)
4	5 (5)	1 (1)	1–2 (2)	8–8 (8)
5	5 (5)	1 (1)	1–2 (1)	7–10 (8)
6	7–8 (7)	1 (1)	2–3 (2)	15–20 (20)
7	5 (5)	1 (1)	2–3 (2)	8–10 (8)
8	5 (5)	1 (1)	2 (2)	0–11 (9)
9	7–8 (7)	1 (1)	2–3 (3)	16–20 (18)
10	5 (5)	1 (1)	2–3 (3)	8–10 (8)
11	5–6 (5)	1 (1)	2–3 (2)	6–10 (9)
12	6–7 (7)	0/1 (1)	2–3 (3)	15–20 (17)
13	4–5 (5)	0/1 (1)	1–2 (2)	7–8 (7)
14				15–18 (16)
15	4–7 (5)	0/1 (1)	1–2 (2)	8–14 (14)
16				10–14 (14)
17				10–14 (14)

***Tergites*.** Tergite 1 reduced, with 3+3 subequal setae (Fig. 4D). Tergite 2 complete, with 2 triangular posterior processes, 5 or 6 lateromarginal setae, 1 inserted seta, 1 central seta (Table 5), anterolateral setae 0.7–0.8 of length of process, processes 1.1–1.2 times as long as broad, basal distance between processes 1–1.2 times as long as their length (Fig. 4D, F). Tergite 3 complete, broader and longer than preceding one with ratios of 0.7–0.9, 1.1–1.3, and 1.1–1.3 respectively, 7 or 8 lateromarginal setae (Fig. 4D). Tergite 4 broader than tergite 3, with ratios 1–1.2, 0.9–1, and 1.3–1.9 respectively, 5 lateromarginal setae (Fig. 4G). Chaetotaxy of tergites 5–7, 8–10, and 11–13 similar as tergites 2–4. Pattern of alternating tergite lengths of 2 short tergites followed by 1 long tergite only disrupted at caudal end (Table 6). Tergites 14 and 16 without processes and with 15–18 and 10–14 setae respectively. Tergite 17 with 10–14 setae. Chaetotaxy and measurements of tergites are given in Tables 5 and 6.

**Table 6.** Measurements of tergites and processes of *Symphylellalongispina* sp. nov. (mean ± se, *n* = 6, in μm) (holotype in brackets).

Tergite	Length	Width	Length of processes	Basal width of processes	Basal distance between processes
1	27 ± 3 (25)	126 ± 2 (125)			
2	45 ± 6 (50)	123 ± 10 (130)	32 ± 2 (34)	28 ± 2 (30)	33 ± 3 (33)
3	97 ± 16 (90)	151 ± 8 (155)	34 ± 2 (37)	28 ± 2 (32)	39 ± 2 (40)
4	60 ± 8 (62)	162 ± 9 (170)	31 ± 2 (29)	34 ± 1 (33)	47 ± 6 (50)
5	60 ± 12 (75)	138 ± 5 (140)	35 ± 4 (39)	28 ± 3 (30)	52 ± 5 (50)
6	115 ± 12 (125)	181 ± 17 (192)	39 ± 3 (41)	31 ± 4 (33)	60 ± 4 (63)
7	72 ± 12 (85)	191 ± 10 (202)	38 ± 4 (36)	39 ± 6 (35)	65 ± 7 (75)
8	71 ± 12 (80)	160 ± 9 (170)	35 ± 3 (38)	27 ± 3 (29)	65 ± 5 (70)
9	124 ± 10 (138)	199 ± 6 (205)	37 ± 3 (41)	30 ± 3 (34)	68 ± 7 (75)
10	74 ± 12 (90)	196 ± 17 (207)	36 ± 3 (37)	36 ± 6 (36)	74 ± 5 (80)
11	72 ± 9 (80)	171 ± 5 (175)	35 ± 1 (34)	29 ± 3 (25)	70 ± 6 (78)
12	121 ± 18 (125)	201 ± 14 (217)	37 ± 4 (38)	30 ± 6 (32)	66 ± 7 (70)
13	71 ± 6 (75)	184 ± 16 (207)	29 ± 3 (27)	36 ± 12 (30)	66 ± 9 (72)
14	68 ± 10 (75)	164 ± 8 (175)			
15	99 ± 10 (100)	180 ± 13 (200)	27 ± 2 (30)	26 ± 3 (26)	55 ± 8 (65)
16	71 ± 7 (75)	148 ± 16 (162)			
17	92 ± 9 (92)	131 ± 9 (135)			

***Legs*.** First pair of legs reduced to 2 small hairy cupules, each with 1 long seta (9–11 μm) (Fig. 4H). Basal areas of legs 2–12 each with 3–5 setae. Leg 12 about 0.6–0.8 of head length (Fig. 5I), trochanter 1.3–1.6 times longer than wide (45–50 μm, 32–36 μm), with 6 or 7 subequal setae in total; femur almost as long as wide (25–33 μm, 25–30 μm), with 5 setae, longest dorsal seta 17–20 μm in length, pubescent dorsally, laterally with cuticular thickenings in pattern of scales; tibia nearly 1.3–1.9 times longer than wide (28–40 μm, 21–23 μm), with 6 or 7 setae, longest dorsal one 14–18 μm; tarsus subcylindrical, 3–3.5 times as long as wide (45–48 μm, 13–16 μm) with 6 dorsal setae: 4 straight and protruding, 2 slightly curved and depressed, longest setae (14–17 μm) about same length of greatest width of podomere, 2 ventral setae close to claw and distinctly shorter than dorsal ones. Claws curved, anterior one broader than posterior one. All legs covered with dense pubescence except areas with cuticular thickenings.

***Coxal sacs*** present at bases of legs 3–9, fully developed, each with 4 setae on surface. Corresponding area of leg 2, 10, 11 and 12 replaced by 1–3 setae (Fig. 4I).

***Styli*** present at base of legs 3–12, subconical (length 5 μm, width 3 μm), basal part with straight hairs; distal quarter hairless and with blunt apex (3 μm) (Fig. 4I).

***Sense calicles*** with smooth margin around pit. Sensory seta inserted in cup center, extremely long (115–120 μm).

***Cerci*** about half length of head, 3.3–3.8 times as long as its greatest width (108–115 μm, 30–34 μm), sparsely covered with 33–39 subequal setae (Fig. 5F, G). Two types of setae inserted on cercus: 4 or 5 long and erect setae located in outer side, and others slightly curved and depressed. Longest outer seta (20 μm) 0.6–0.7 of greatest width of cerci, terminal area short (16–18 μm), circled by 6–8 layers of curved ridges. Terminal setae (15–16 μm) slightly shorter than terminal area (Fig. 5F, G).

##### Etymology.

The species name is derived from the Latin words “*longus*” and “*spina*” meaning “long spine”. It is feminine and refers to the extremely long proximal spines on the pars molaris of the mandible.

##### Distribution.

Known only from the type locality.

##### Remarks.

*Symphylellalongispina* sp. nov. has a thickened and prominent labrum and irregular bladder-shaped organs on antennae, which separate it from all other congeners. It is most similar to *S.asiatica* Scheller, 1971 from India and Sri Lanka in the shape and chaetotaxy of the tergites, but the new species differs in the distal part of the processes (distinctly swollen in *S.longispina* sp. nov. vs small and slender in *S.asiatica*), in the shape and chaetotaxy of cerci (subcylindrical and with sparse setae in *S.longispina* sp. nov. vs conical and with dense setae in *S.asiatica*), and in the shape of the palp of the first maxilla (slightly curved in *S.longispina* sp. nov. vs straight in *S.asiatica*). The new species is also similar to *S.brincki* Scheller, 1971 from Sri Lanka in the chaetotaxy of the tergites, but they can be easily separated by the central rod (with a middle node-like interruption in *S.longispina* sp. nov. vs with a narrow transverse interruption in *S.brincki*), by the end of the processes (with round end-swellings in *S.longispina* sp. nov. vs spatulate end-swellings in *S.brincki*), and by the shape and chaetotaxy of cerci (3.3–3.8 times as long as wide and with sparse setae in *S.longispina* sp. nov. vs 2.3 times as long as wide and with dense setae in *S.brincki*).

## ﻿Discussion

*Symphylella* is one of the most common and diverse group of symphylans with a wide global distribution (Szucsich and Scheller 2011; Bu and Jin 2018). The central rod on the head, the Tömösváry organ, the processes of tergites, the stylus, and the cercus are commonly used as diagnostic characters for species of this genus and, thus, were previously described and illustrated in detail (Scheller 1971; Szucsich and Scheller 2011). However, in recent years, we have found that some of characters, such as the first maxilla, the mandible, and the head chaetotaxy, are differ among species and good for species diagnosis (Jin and Bu 2018, 2019, 2020; Jin et al. 2019), but they were often overlooked by former specialists.

The mandible structure of Symphyla was carefully studied and compared with other arthropods by former colleagues (Richter et al. 2002; Edgecombe et al. 2003). According to their excellent scanning electron photomicrographs, the mandibular gnathal edge of *Hanseniella* (Scutigerellidae) is composed of the pars incisivus (pi) and pars molaris (pm), with lacinia mobilis inserted between. We have observed the similar parts in the species of *Symphylella* (Scolopendrellidae) using light microscopy, but the shape and composition of each part are different to that of *Hanseniella*. The structure of mandible is varied among species of *Symphylella*, which can be diagnostic character of species. To obtain a better perspective overall of mandible structures in Symphyla, the study of more species using SEM method is needed.

**Figure 6.**
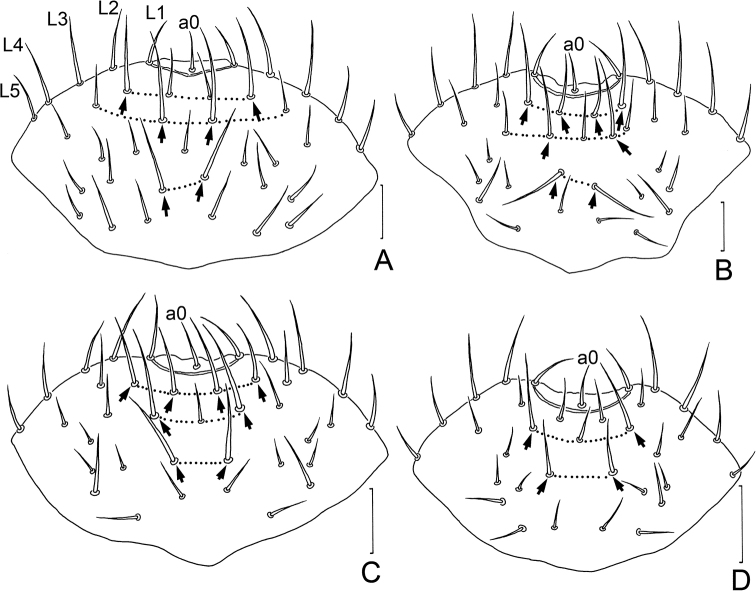
Frons of *Symphylella* spp. from China. **A***S.macropora***B***S.zhongi***C***S.communa***D***S.minuta*. Scale bars: 20 μm. (L1–L5 – lateral setae, a0 – antero-central seta, arrows indicate macrosetae).

In our study of *Symphylella* specimens from Zhejiang and Shanghai, we observed that the extremely long setae on the frons of *S.macrochaeta* sp. nov. differ from other Chinese congeners (Fig. 3H). Thus, we checked the other four species recorded in China and compared their frons chaetotaxy (Fig. 6A–D). As a result, we confirmed that the frons chaetotaxy is a useful diagnostic character in the taxonomy of *Symphylella* (Table 7).

According to our observations, the frons of *Symphylella* spp. often has well-differentiated macrosetae located on the 2/3 anterior part and 5+5 setae on the lateral margin. The quantity, length, arrangement, and ratio to antero-central seta of the macrosetae vary among species but vary little among conspecific individuals (Table 7). A broader study to reexamine the type materials of all other described species of *Symphylella* is needed to supplement the missing data.

**Table 7.** Comparison of chaetotaxy on frons of *Symphylella* spp. from China.

Characters	*S.macrochaeta* sp. nov.	*S.longispina* sp. nov.	*S.macropora*	*S.zhongi*	*S.communa*	*S.minuta*
Number of macrosetae (M)	10	8	6	8	8	4
Formula of M-setae	4/4/2	4/2/2	2/2/2	4/2/2	4/2/2	0/2/2
length of M-setae (μm)	58–73	21–28	25–37	22–37	20–30	12–20
length of a0 setae (μm)	12–15	7–8	12	12–15	10–16	7–11
M/a0	4–5.6	2.8–3.7	2–3	1.5–2.7	1.6–2.7	1.4–2.4

## Supplementary Material

XML Treatment for Symphylella

XML Treatment for Symphylella macrochaeta

XML Treatment for Symphylella longispina
